# Response of Soil Microbial Communities Between Different Vegetation Types in the Greater and Lesser Khingan Mountains Ecotone in Northeast China

**DOI:** 10.3390/microorganisms13092107

**Published:** 2025-09-09

**Authors:** Weiping Yin, Xinmiao Guo, Dalong Ma, Huan Yu

**Affiliations:** 1 College of Geographical Sciences, Harbin Normal University, Harbin 150025, China; 2024400071@stu.hrbnu.edu.cn (W.Y.); guoxinmiao2003@163.com (X.G.); yuhuan10729@163.com (H.Y.); 2 Institute of Industrial Crops, Heilongjiang Academy of Agricultural Sciences, Harbin 150086, China

**Keywords:** ecotone, diversity, forest types, soil microbial community, restoration

## Abstract

The Greater and Lesser Khingan Mountains ecotone exhibits transitional characteristics between northern coniferous forests and cold-temperate mixed coniferous-broadleaf forests. However, it remains unknown how vegetation characteristics and soil properties jointly influence the structural patterns of soil microbial communities within the forest systems of this ecological transition zone. Therefore, we used high-throughput sequencing and soil physicochemical analysis to investigate the response mechanisms of soil microbial communities during forest succession (five representative natural secondary forests) and their environmental driving factors. The results showed that the bacterial communities in the Greater and Lesser Khingan Mountains ecotone were primarily dominated by Proteobacteria, Acidobacteriota, Actinobacteriota, Chloroflexi, and Verrucomicrobiota, while the fungal communities were primarily dominated by Basidiomycota and Ascomycota. Their relative abundances exhibited significant differences dependent on vegetation types. Different vegetation types significantly influenced the α-diversity of soil microbial communities at the study site; however, there were no significant differences in microbial α-diversity except for the bacterial Chao 1 index. The pH, NO_3_^−^-N, NH_4_^+^-N, MAOC, TN, TP, and DOC significantly influenced bacterial communities, while DOC and pH were the key environmental factors shaping soil fungal communities. This study reveals the synergistic succession patterns among vegetation, soil, and microorganisms, providing a microbiological basis for high-latitude cold-region forest restoration under climate change.

## 1. Introduction

As the main component of terrestrial ecosystems, forests are vital for preserving biodiversity and regulating the global climate [1,2]. In forest ecosystems, soil microorganisms constitute an important functional component [3], dominating litter decomposition and core biogeochemical cycles [4,5,6], and serving as the core driving force for maintaining the structure, function, and stability of forest ecosystems [7,8]. Notably, dynamic changes in forest vegetation types significantly drive the evolution of soil microbial community structure, functional activity, and diversity patterns [1]. A long-term coevolutionary relationship has been established between plants and soil microorganisms: vegetation provides organic matter as a primary carbon source to sustain microbial survival, while microorganisms decompose over 90% of litter, mineralizing organic matter and releasing it as inorganic nutrients available for plant uptake [9]. Consequently, deciphering the spatiotemporal dynamics of soil microbial communities and their drivers holds critical mechanistic importance for predicting how climate change, disturbances, and human activities impact forest ecosystem resilience.

There is ample evidence to suggest that the evolution of soil physical and chemical properties, plant diversity, and community composition are key ecological drivers shaping the structure, function, and diversity of soil microbial communities [7,8]. Within homogeneous climatic zones, vegetation type is a critical factor regulating soil microbial community composition [10], primarily due to the significant differences in the chemical characteristics of plant litter and root exudate components/fluxes between different vegetation types, which directly influence the colonization and development of soil microorganisms [11]. However, studies on the association between forest vegetation types and soil microbial community structure and diversity exhibit complexity and even contradictions. For example, some studies have found that soil microbial abundance in broadleaf forests is typically significantly higher than in coniferous forests [12], and that changes in forest type may lead to significant shifts in microbial community structure and diversity [13]. However, the results of Chen et al. [14] are contrary to this, as although the microbial community structures of four tropical rainforest soils were significantly differentiated, their Shannon diversity indices and species richness did not show statistically significant differences. More complexly, Yang et al. [15] found in their study of the Greater Khingan Range that even in coniferous forests dominated by the same dominant tree species (Larix), differences in understory vegetation composition can lead to significant differences in soil microbial Shannon indices and richness. As Sui et al. [16] found, the driving factors behind differences in soil bacterial and fungal community structures during early succession differ from those in later succession. Soil microbial structure is primarily determined by soil properties rather than plant communities. We believe these seemingly contradictory findings may arise because driving factors exhibit distinct modes of action across biotic communities, forest stands, and microhabitats; dominant drivers of microbial community assembly may vary along succession trajectories; and differing research focuses (bacteria vs. fungi), sequencing depths, or emphasis on structural versus diversity metrics may yield divergent results. Resolving these inconsistencies requires studying microbial dynamics during specific succession stages within distinct, ecologically diverse ecosystems, while explicitly accounting for the synergistic changes in soil properties and vegetation characteristics. The ecological transition zone between the Greater Khingan and Lesser Khingan Mountains provides an ideal model system for this purpose.

As the unique ecological transitional zone in northeastern Eurasia, the Greater and Lesser Khingan Mountains ecotone features the transitional characteristics of both the northern coniferous forests and the cold temperate mixed coniferous and broadleaf forests, and it is also a sensitive area that responds to climate change and human activities. *Larix gmelinii* is the climax forest ecosystem, but excessive exploitation of this resource over the past 100 years has caused significant landscape changes. Larch forests have severely degraded into other vegetation types, and most of the current forests are naturally regenerated secondary forests at different successional stages, including mixed coniferous and broadleaf forests and broadleaf forests. This provides an excellent opportunity to assess how vegetation characteristics and soil properties jointly influence the structural patterns of soil microbial communities in the forest systems of the Greater and Lesser Khingan Mountains ecotone. In this study, we used high-throughput sequencing methods to analyze the diversity and composition of soil microbial (bacterial and fungal) communities and posed the following scientific questions: (i) How do soil microbial communities respond to forest succession? and (ii) Which soil characteristics are the primary determinants of microbial diversity and community structure? In order to answer these questions, we selected five typical natural secondary forests (QM: Mongolian oak forest, BP: white birch forest, QB: mixed Mongolian oak and white birch forest, LB: mixed larch and white birch forest, LG: larch forest) as experimental subjects. We investigated the variations of soil bacterial and fungal communities in these five typical forests. We hypothesized that (1) the α- and β-diversities and compositions of soil microbial communities differ between different forests, and (2) the driving mechanisms of soil microbial communities change with different forests.

## 2. Materials and Methods

### 2.1. Study Site

**Figure 1:**
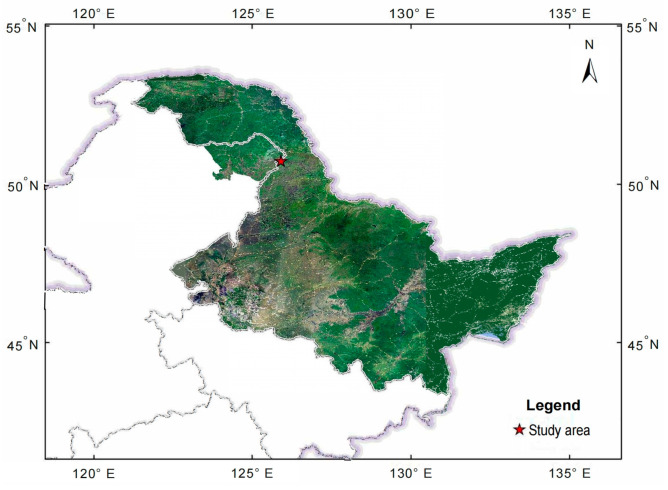
Location of the study sites.

The experimental site is located at the southern foothill of Yilehuli Mountain in the Greater and Lesser Khingan Mountains ecotone (50°47′24′′–50°48′10′′ N, 125°55′58′′–125°56′21′′ E) (Figure 1). This region has a cold-temperate continental monsoon climate, characterized by long and severe winters and short and warm summers, with an annual average temperature of −0.4 °C, extreme winter temperatures reaching −48 °C, and summer temperatures peaking at 37 °C. The soil freezing period and the snow cover period last representively about 270 and 200 days annually, while annual precipitation averages 450–550 mm, and the maximum depth of the soil permafrost layer is 250 cm. The above-ground vegetations of five different forests distributed randomly due to the heterogeneity of topography and soil nutrients, another reason that the human activities such as logging, burning, and forest management resulted the vegetation distribution randomly. Forest types were coniferous forests, mixed coniferous and broadleaf forests, and broadleaf forests [17]. The dominant species are mainly *Larix gmelinii*, *Betula platyphylla*, *Betula dahurica*, *Quercus mongolica*, *Populus davidiana*, *Tilia amurensis*, *Chosenia arbutifolia*, *Prunus padus*, *Salix raddeana*, and *Alnus mandshurica*.

### 2.2. Experimental Design and Soil Sample Collection

Five typical natural secondary forests (QM: Mongolian oak forest, BP: white birch forest, QB: mixed Mongolian oak and white birch forest, LB: mixed larch and white birch forest, LG: larch forest) were selected as experimental subjects within the Greater and Lesser Khingan Mountains ecotone. Within each forest type, we established three plots (approximately 400 m^2^ each). Each plot contained 3 nested subplots measuring 10 m by 10 m, positioned at the diagonals and center point, totaling 45 subplots.

Surface soil samples (0–10 cm depth) were collected in September 2024 using a 5-cm diameter soil auger after removing surface debris (e.g., leaves and dry vegetation). Samples from the three subplots within each individual plot were composited (i.e., mixed together), resulting in one composite sample per plot.

Composite plot samples were placed in self-sealing bags and stored at 4 °C immediately after collection. Samples were transported to the laboratory promptly. Upon arrival, each composite sample was homogenized by sieving through a 2 mm mesh. A portion of the homogenized sample was air-dried for analysis of soil physical and chemical properties. The remaining sample was stored at −80 °C for microbial community analysis.

### 2.3. Soil Physicochemical Properties Analyses

Soil physicochemical properties, including pH, soil moisture content (SWC), ammonium nitrogen (NH_4_^+^-N), nitrate nitrogen (NO_3_^−^-N), total nitrogen (TN), total phosphorus (TP), total carbon (TC), and dissolved organic carbon (DOC), were determined using the methods described in our previous studies [18,19]. Particulate organic carbon (POC) and mineral-associated organic carbon (MAOC) were measured using an elemental analyzer (Elementar Vario EL III, Langenselbold, Germany).

### 2.4. DNA Extraction, Metagenome Sequencing, and Data Processing

Soil microbial genomic DNA was extracted using the DNeasy PowerSoil Pro Kit (Qiagen, Hilden, Germany). A 0.25 g fresh soil sample was weighed, and cells were disrupted using a bead milling method (0.1 mm glass beads vortexed for 10 min) combined with a dedicated fungal cell wall lysis step using Lyticase enzyme pretreatment (3.0 KU mL^−1^, 37 °C for 30 min; Sigma-Aldrich, St. Louis, MO, USA). This enzymatic step was implemented to enhance the disruption of recalcitrant fungal cell walls composed primarily of chitin, which are less susceptible to mechanical breakage alone [20]. After two rounds of centrifugation to remove humic acid inhibitors, DNA was purified using a silica membrane adsorption column and eluted with ultra-pure water. DNA quality was assessed using a spectrophotometer (NanoDrop Lite, Wilmington, DE, USA). The V3-V4 region of the bacterial 16S rRNA gene was amplified using primers 338F/806R, and the ITS region of fungi was amplified using primers ITS1F/ITS2R. The amplified products were purified using AMPure XP magnetic beads, and a double-ended indexed library was constructed using the Nextera XT Index Kit. Fragment sizes were verified using the Agilent 2100 Bioanalyzer (Agilent, Santa Clara, CA, USA). Sequencing was performed on the Illumina MiSeq platform (PE300) (Illumina, San Diego, CA, USA), yielding approximately 50,000 high-quality reads per sample. Raw data were filtered for low-quality sequences (Phred score < 20) using Trimmomatic and assembled using FLASH. Bacterial ASVs were generated using DADA2 (annotated with the SILVA v138 database), and fungal ASVs were generated using UNITE v8.3.

### 2.5. Statistical Analysis

All statistical analyses were performed in the R environment (v4.3.0) [21], primarily relying on the following packages: data preprocessing and analysis of variance (ANOVA) used the stats package (v4.3.0), and multivariate analysis employed the vegan package (v2.6) [22]. Multiple comparison tests were performed using the agricolae package (v1.3) [23], and graphics were generated using ggplot2 (v3.4.4) [24] and VennDiagram (v1.7.3) [25]. Soil physicochemical parameters were first subjected to Shapiro-Wilk normality tests and Levene’s tests for variance homogeneity; differences in parameters between forest types were analyzed using one-way ANOVA, with significant results subjected to Tukey HSD post hoc tests and labeled with letters. Differences in microbial α-diversity indices across forest types were assessed using one-way Analysis of Variance (ANOVA). This parametric approach was justified as all tested diversity metrics met the assumptions of normality (Shapiro-Wilk test, *p* > 0.15) and homogeneity of variances (Levene’s test, *p* > 0.10). Where ANOVA indicated significant differences (*p* < 0.05), Tukey’s Honest Significant Difference (HSD) post hoc tests were applied for pairwise comparisons; β diversity was analyzed using the vegan: metaMDS function based on Bray-Curtis distance for non-metric multidimensional scaling (NMDS, 999 permutations), and the significance of community differences was validated using ANOSIM. Microbial community structure was analyzed using hierarchical clustering (Euclidean distance, Ward.D2 algorithm) and Venn diagrams. Pearson correlations between α diversity and environmental factors were calculated using Hmisc:rcorr (v4.7) [26]. Microbial community-environment factor relationships were quantified using Redundancy Analysis (RDA), and the significance of constrained axes was assessed via 999 permutations.

## 3. Results

### 3.1. Physicochemical Properties of Soils in Different Forest Types

All soil physicochemical properties differed significantly in different forest types (Table 1, *p* < 0.05), except MAOC. The soil pH was highest in QM and significantly lower in QB, LB, and LG compared to QM and BP (*p* < 0.05). The concentrations of SWC, NH_4_^+^-N, NO_3_^−^-N, TN, TP, TC, and POC in soils were highest in LG (*p* < 0.05). The concentrations of MAOC in soils were highest in LB, lowest was found in BP (*p* < 0.05).

### 3.2. Soil Microbial Community Diversity in Different Forest Types

**Figure 2:**
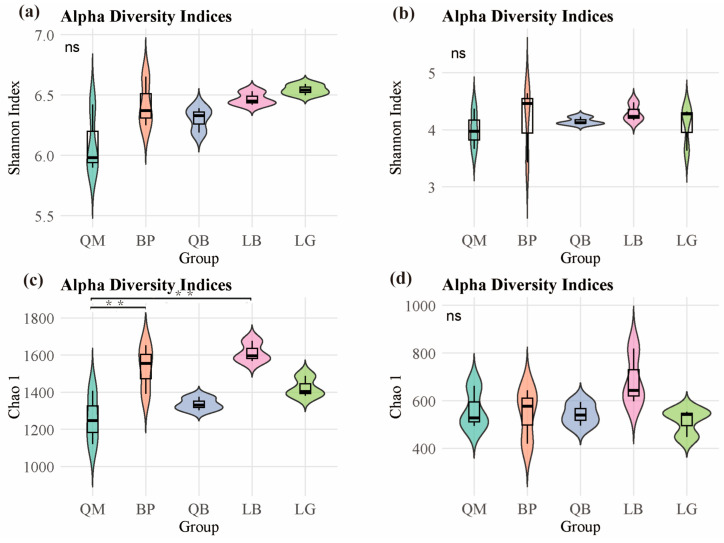
α-diversity of soil microbial communities in different forest types: (**a**,**c**) bacterial communities (*n* = 3); (**b**,**d**) fungal communities. The asterisk indicates significant differences (** *p* < 0.01; Tukey test). ‘ns’ indicates no significant difference. QM: Mongolian oak forest, BP: white birch forest, QB: mixed Mongolian oak and white birch forest, LB: mixed larch and white birch forest, LG: larch forest.

**Figure 3:**
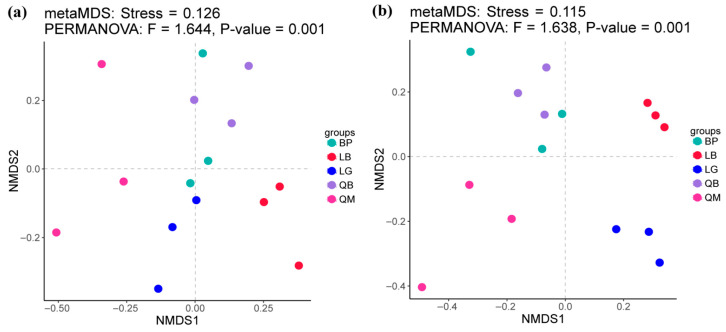
NMDS analysis of bacterial (**a**) and fungal (**b**) communities in soils of different forest types. QM: Mongolian oak forest, BP: white birch forest, QB: mixed Mongolian oak and white birch forest, LB: mixed larch and white birch forest, LG: larch forest.

The results showed that there were no significant differences in the Shannon index of bacterial communities in soils of different forest types (Figure 2a). However, as shown in Figure 2c, the Chao 1 index of bacterial communities in BP and LB soils was significantly higher than that in QM soils (*p* < 0.01). However, there were no significant differences in the Shannon index and Chao 1 index of fungal communities in soils of different forest types (Figure 2b,d). NMDS analysis results showed that each forest type was associated with unique soil microbial community, there were significant differences in the β-diversity of soil bacterial communities (PERMANOVA, F = 1.644 and *p* < 0.05) and fungal communities (PERMANOVA, F = 1.638 and *p* < 0.05) between different forest types (Figure 3).

### 3.3. Composition of Soil Microbial Communities in Different Forest Types

**Figure 4:**
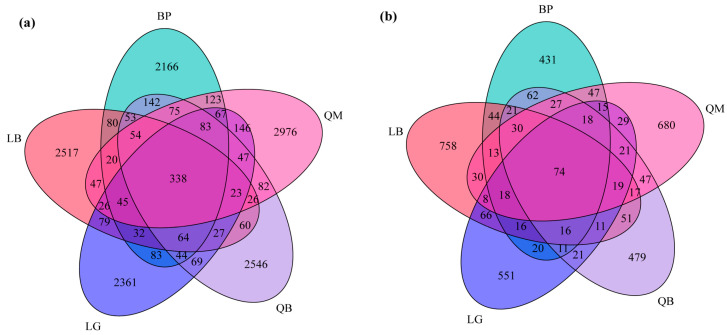
Venn diagrams showing the shared and unique ASVs of soil bacteria (**a**) and fungi (**b**) between different forest types.

The structure of soil microbial communities, particularly the unique and shared ASVs in the samples, was visualized using Venn diagrams (Figure 4). Each forest type soil bacterial community contained a large number of unique ASVs: LB had 2517, BP had 2166, QM had 2976, QB had 2546, and LG had 2361. The five forest types shared 338 bacterial ASVs (Figure 4a). In terms of soil fungi, LB had 758 unique ASVs, BP had 431, QM had 680, QB had 479, and LG had 551. There were 74 fungal ASVs shared among the five forest types (Figure 4b).

**Figure 5:**
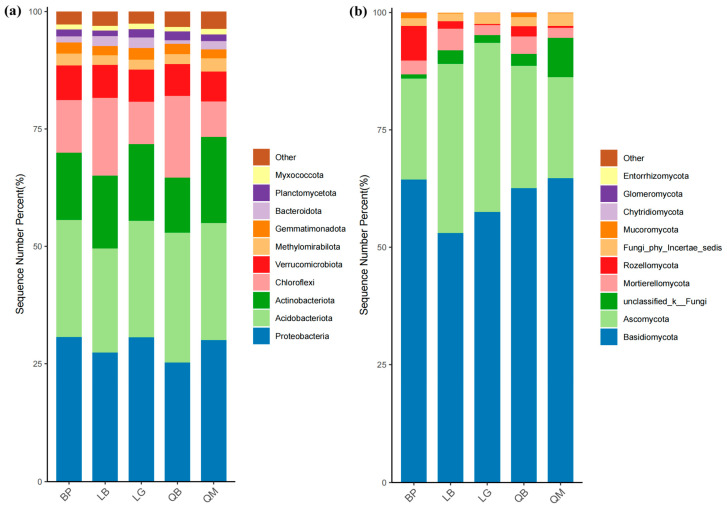
Differences in bacterial and fungal community composition among different forest types. Composition of bacteria (**a**) and fungi (**b**) in soil at the phylum level.

The stacked bar chart showed the composition of the top 10 bacterial and fungal phyla in terms of abundance (Figure 5). There were significant differences in the distribution of microbial phyla among the five different forest types (Tables S1–S4). As shown in Figure 5a and Table S1, Proteobacteria dominated in BP, LG, and QM, but had the lowest relative abundance in QB. In contrast, Actinobacteria reached a peak in the QM group, higher than in other forest types. Chloroflexi exhibited significantly higher relative abundances in LB and QB compared to other forest types (*p* < 0.01). As shown in Figure 5b and Table S3, Basidiomycota dominated in all forest types, followed by Ascomycota. Notably, Basidiomycota accounted for over 64% of the relative abundance in BP and QM, and the relative abundance of Basidiomycota in LB was significantly lower than in other forest types (*p* < 0.01). A few phyla (such as Rozellomycota in BP and Mortierellomycota in LB) exhibited specific variations across different groups, but overall accounted for less than 5% of the community.

**Figure 6:**
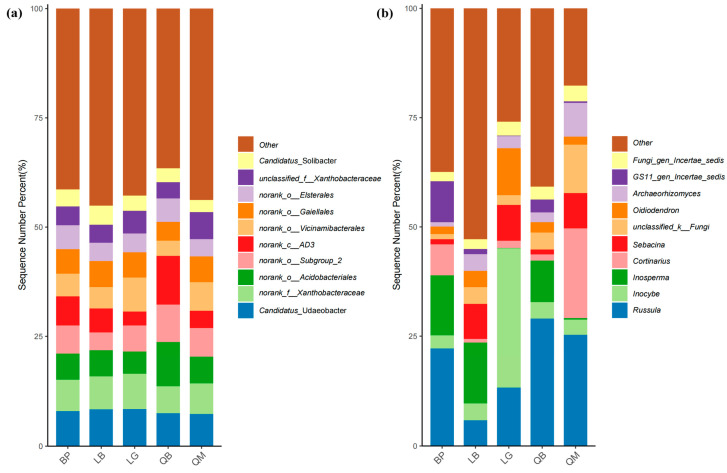
Differences in bacterial and fungal community composition among different forest types. Composition of bacteria (**a**) and fungi (**b**) in soil at the genus level.

*Candidatus*_Udaeobacter, Xanthobacteraceae, o_Acidobacteriales, o_Subgroup_2, *c_AD3*, o_Vicinamibacterales, and o_Gaiellales were the taxa with the highest relative abundances in bacterial communities across different forest types (Tables S5–S8). As shown in Figure 6a and Table S6, *Candidatus*_Udaeobacter exhibited the highest relative abundance in BP, while *c_AD3* and o_Acidobacteriales showed significantly higher relative abundances in QB compared to other forest types (*p* < 0.01). *Russula* dominated in BP, QB, and QM, but the most abundant fungal genera in LB and LG were *Inosperma* and *Inocybe*, respectively (Figure 6b, Tables S7 and S8). Notably, some fungal genera exhibited relatively high relative abundances in certain forest types but were detected at extremely low levels in others (e.g., *GS11_gen_Incertae_sedis* in BP and *Tomentella* in LB).

**Figure 7:**
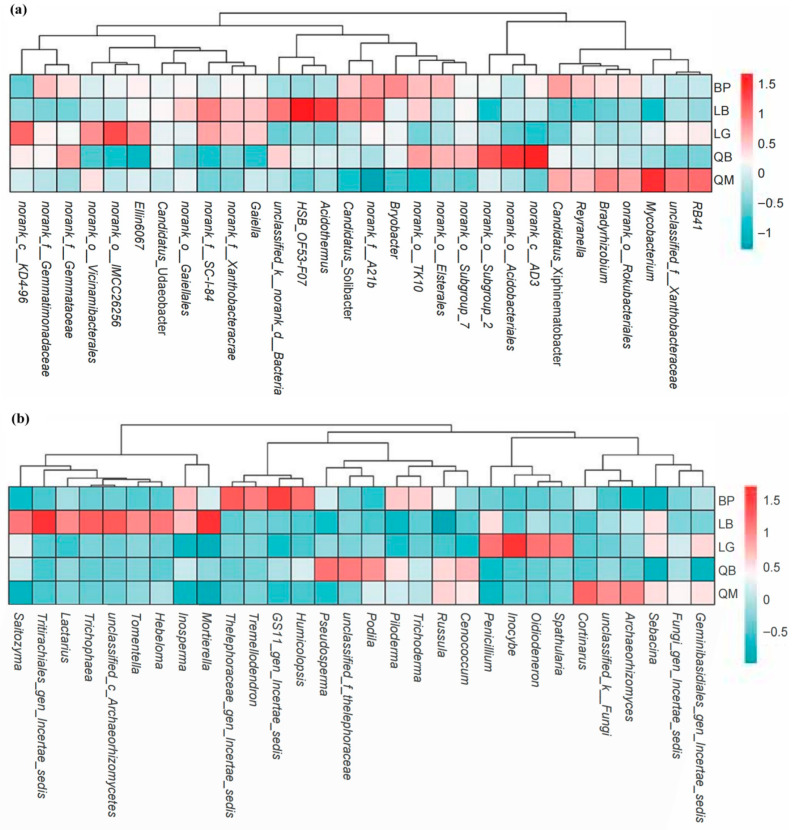
Heat map and hierarchical clustering of the relative abundance of the top 30 genera detected in soil bacterial (**a**) and fungal (**b**) communities. The color gradient (red, white, blue) indicates the relative abundance of soil microorganisms from high to low in different forest types.

The cluster heat map showed the top 30 genera detected in soil bacterial (a) and fungal (b) communities in terms of relative abundance. We found that the soil fungal community structure of different forest types had more obvious group specificity than that of bacteria (Figure 7).

### 3.4. Main Shapers of Soil Microbial Community Structure

According to Pearson correlation analysis (Table 2), the bacterial Shannon index was positively correlated with soil TN (r = 0.531, *p* < 0.05) and TC (r = 0.624, *p* < 0.05) values, while the bacterial Chao 1 index was negatively correlated with DOC (r = −0.693, *p* < 0.01). Additionally, the fungal Chao 1 index was negatively correlated with DOC (r = −0.554, *p* < 0.05).

**Figure 8:**
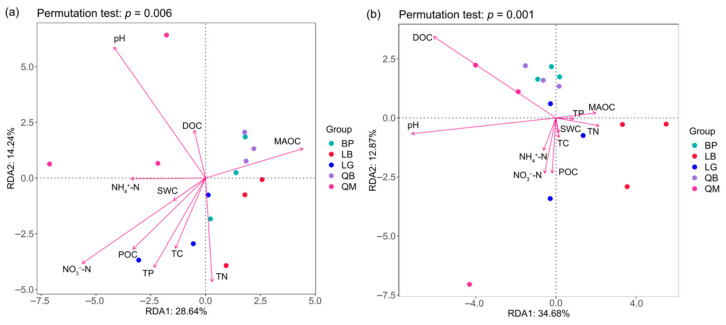
RDA analysis of soil physicochemical properties and soil bacteria (**a**) and fungi (**b**) in different forest types.

Redundancy analysis (RDA) indicated that soil properties were key environmental factors shaping soil microbial communities (Figure 8). As shown in Figure 8a, pH, NH_4_^+^-N, NO_3_^−^-N, MAOC, TN, TP, and DOC significantly influenced bacterial communities (*p* < 0.05). QM was negatively correlated with NO_3_^−^-N, TN, and TP, but positively correlated with pH and DOC. QB was closely correlated with MAOC, while LB and LG were primarily driven by NO_3_^−^-N, TN, and TP, and BP was significantly negatively correlated with pH. As shown in Figure 8b, DOC and pH were the key environmental factors shaping the soil fungal community and were significantly negatively correlated with LB (*p* < 0.05).

**Figure 9:**
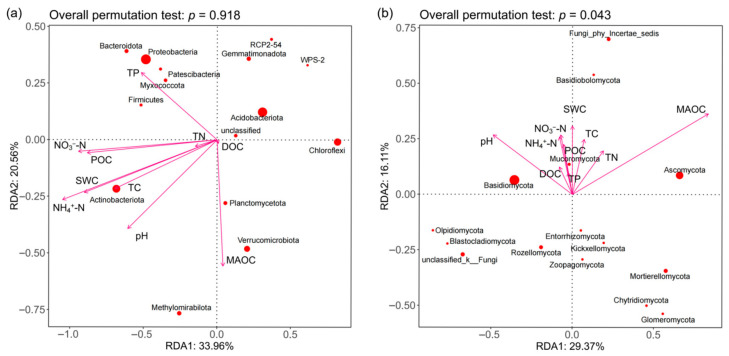
RDA of dominant bacteria (**a**) and fungi (**b**) in soil constrained by soil variables.

For bacterial communities (Figure 9a), the RDA model explained 54.52% of the total variance (unadjusted R^2^ = 0.545); however, after adjusting for model complexity and sample size, the explanatory power decreased to 32.1% (adjusted R^2^ = 0.321). The overall effect of environmental variables on the bacterial community did not reach statistical significance (F = 0.26, *p* = 0.918, 1000 permutations), suggesting that even the adjusted R^2^ value may reflect overfitting or random noise rather than true biological associations. The key environmental drivers including carbon-related factors (DOC, TC, POC, MAOC) and nitrogen compounds (TN, NO_3_^−^-N, NH_4_^+^-N). Bacterial phyla exhibited distinct environmental preferences: Proteobacteria was associated with TP, Verrucomicrobiota was closely related to MAOC, and Actinobacteriota aligned with moisture and NH_4_^+^-N and TC. For fungal communities (Figure 9b), the RDA model was statistically significant (F = 2.84, *p* = 0.043, 1000 permutations), with RDA1 and RDA2 explaining 29.37% and 16.11% of the total variance, respectively (cumulative unadjusted R^2^ = 0.4548). After adjustment, the model retained 28.7% explanatory power (adjusted R^2^ = 0.287), indicating a robust albeit moderate influence of environmental variables on fungal phyla. MAOC was significantly correlated with fungal phyla Ascomycota (Figure 9b, *p* < 0.05).

## 4. Discussion

### 4.1. Differences in the Physicochemical Properties of Soils in Different Forest Types

This study reveals differences in soil physicochemical properties among forest types. Specifically, the higher pH values in Mongolian oak forest (QM) may be attributed to the alkaline ash elements in their litter neutralizing soil acidity during decomposition [27]; whereas the lower pH values in coniferous forests (LG, LB) are associated with the release of organic acids and higher cation absorption by coniferous litter [28]. The higher SWC, nitrogen and phosphorus content (TN, TP), and carbon pool components (TC, POC) in larch forest (LG) compared to other forest types reflect the stronger ability of coniferous forest canopies to intercept precipitation, as well as the high lignin content in their litter, which slows decomposition and promotes the accumulation of surface organic matter [29]. Similarly, even under the same climatic conditions, variations in litter characteristics produced by different tree species are associated with differences in soil quality [30]. Notably, the MAOC content in mixed larch and white birch forest (LB) was higher than in other types, white birch forest (BP) had the lowest MAOC, indicating that single broadleaf forests have weaker capacity to maintain mineral-protected carbon pools. Some studies have pointed out that MAOC content increases with the proportion of plant mixing, and mixed forests promote the stability of mineral-bound carbon through the input of litter diversity and enhanced microbial activity [31]. These results confirm the core role of forest types in driving the differentiation of soil carbon and nitrogen cycles by altering litter chemical properties and microenvironments [32].

### 4.2. Differences in Soil Microbial Community Diversity and Composition Among Different Forest Types

This study observed significant microbial community differentiation in the response of soil microbial diversity across different forest types. Mixed forests enhance bacterial diversity by increasing the colonization of rare species rather than altering the evenness of dominant species [33]. In contrast, the intriguing characteristics and compositional specificity of fungal community diversity confirm the high sensitivity of fungal communities to environmental selection. In particular, variations in leaf chemical properties (such as lignin/N ratio) and microenvironments (such as pH, moisture content) drive the specific distribution of fungal functional groups [34]. Studies have shown that coniferous forests enrich acid-tolerant fungi, while broadleaf forests prefer cellulose-degrading fungi [35]. Additionally, the similarity between BP and QB fungal communities may stem from similar C/N ratios and secondary metabolites in their litter, while LB’s mixed coniferous and broadleaf litter shapes unique fungal communities through resource heterogeneity [34,36]. We found that the proportions of fungal ASVs shared across the five forest types were significantly lower than that of bacteria (Figure 4), indicating that fungal community assembly exhibited stronger habitat specificity, while bacterial communities demonstrated broader ecological niche adaptability. Bacteria, with their metabolic flexibility and rapid dispersal ability, can adapt their core communities to various resource conditions, enabling them to share a higher proportion of ASVs across different forest types [37]. Additionally, fungal host specificity (e.g., the symbiotic relationship between ectomycorrhizal fungi and specific tree species) further limits their distribution across habitats, exacerbating the divergence in shared ASV proportions between the two groups [38]. Specifically, the phylogeny within plant-arbuscular mycorrhizal symbiosis exhibits conservation, forming a “taxonomic corridor” that filters regional species pools. The high-energy-cost growth characteristics of mycelial hyphae drive their preferential colonization of compatible hosts, and the resulting preferential effects exacerbate community differentiation.

We used stacked bar charts to illustrate the significant differences in the distribution of five different forest types at the phylum and genus levels of microorganisms. The dominance of Proteobacteria in BP, LG, and QM may be related to its metabolic diversity (Figure 5a). This phylum contains a large number of chemotrophic heterotrophs involved in carbon and nitrogen cycles, and its abundance changes may be driven by the chemical properties of litter and soil physical properties [39]. The peak abundance of the Actinobacteriota phylum in QM may be associated with the high tannin content of Mongolian oak litter, which is characteristic of this forest type. According to previous studies, tannins can promote the colonization of Actinobacteriota (such as Streptomyces) by inhibiting other bacterial groups [40]. The high abundance of Chloroflexi in LB and QB suggests ecological niche differentiation of oligotrophic bacteria in mixed forest soils, with such bacterial communities potentially adapting to carbon sources formed by mixed coniferous and broadleaf litter [39]. The absolute dominance of the Basidiomycota phylum in fungal communities (Figure 5b) and the absolute dominance of the *Russula* genus (Figure 6b) are closely related to lignin degradation functions. Chen et al. [41] found that in forest ecosystems, Basidiomycota containing laccase genes dominate the litter layer, and their community structure is significantly correlated with soil pH, C/N ratio, and guaiacol content in lignin. However, the abundance of Basidiomycota in LB was significantly reduced, possibly due to the input of coniferous litter from the mixed larch and white birch forest, which is rich in resin acids and other secondary metabolites, potentially inhibiting certain Basidiomycota while promoting the growth of stress-tolerant groups such as *Inosperma* [42]. The specific distribution of low-abundance phyla such as Rozellomycota and Mortierellomycota further confirms the screening effect of microenvironmental heterogeneity on rare fungal groups [40]. At the fungal genus level, the enrichment of *Candidatus*_Udaeobacter in BP was closely related to its metabolism of birch root exudates (Figure 6a). Some studies have reported that certain Acidobacteriota phylum taxa can gain metabolic advantages by degrading mannitol [43]; while the high abundance of *norank_c_AD3* (Acidobacteriota class) and *norank_o_Acidobacteriales* in QB (Figure 6a) corresponds to the low pH and high DOC of this forest type, consistent with other studies [44,45].

### 4.3. Soil Microbial Community Diversity and Composition Are Related to Soil Physicochemical Properties

We found that the bacterial Shannon index was significantly positively correlated with soil TN and TC (Table 2), which is consistent with the findings of Liu et al. [46]. Kim et al. [47] found that the absolute abundance of Acidobacteriota was highly positively correlated with organic matter content. However, the negative effect of DOC on the bacterial Chao 1 index in our results was the opposite of theirs, which may be due to differences in the chemical composition of forest litter, especially when DOC is rich in phenolic compounds such as tannins, which selectively promote acid-tolerant bacteria (e.g., *norank_c_AD3*) while inhibiting sensitive bacterial communities. Redundancy analysis (RDA) indicated that the explanatory power of the selected environmental variables on bacterial community structure did not reach statistical significance (*p* = 0.918), which contradicted our initial hypothesis. RDA’s reliance on dominant environmental gradients may perform poorly when data noise is high. Future studies should employ biased RDA to control for confounding variables or explore mixed models (e.g., db-RDA combined with PERMANOVA) to enhance statistical power. Additionally, increasing sample sizes and incorporating time-series observations may help capture potential environmental-microbial dynamic couplings. The negative correlation between fungal Chao 1 index and DOC suggested that high DOC may limit fungal diversity, contrasting with the dominance of Basidiomycetes like *Inocybe* in low DOC environments in LG. RDA further revealed that DOC and pH were key drivers of fungal community structure, consistent with findings from coniferous forests-soil pH significantly influences phylum level distribution by screening acid-tolerant fungi (e.g., Mortierellomycota) [42]. Additionally, the positive correlation between MAOC and the Ascomycota phylum may stem from its ability to decompose recalcitrant carbon (Figure 9), a similar finding observed in the regulation of carbon cycling by Acidobacteriota viruses in Arctic tundra soils [48]. Redundancy analysis (RDA) indicated that DOC and pH are key environmental factors shaping soil fungal communities (Figure 8b). This co-evolution of pH and DOC shapes fungal resource allocation patterns [34,49]. Protonation of humic acids enhances metal binding capacity while inhibiting manganese peroxidase activity in white rot fungi. Neutral microenvironments enable the Morcellaceae to hydrolyze ester-linked DOC via chitinase secretion, explaining their pH-dependent distribution patterns. Limitations of RDA analysis (bacterial, *p* = 0.918) indicate that DOC-microbe interactions exhibit finer-tuned regulatory mechanisms at the metabolic level than those revealed by holistic chemical analysis. Future research should employ external metabolomics to link specific leaf fall metabolites with microbial trait trade-offs, and utilize nano-SIMS to quantify isotopic flux of ^13^C-labeled DOC toward ECM hyphae and bacterial biofilms, thereby resolving carbon allocation paradoxes.

This study investigated the microbial structure and diversity of different forest types in the Greater and Lesser Khingan Mountains ecotone, providing data support for understanding changes in soil microorganisms during high-latitude forest vegetation succession, and offering a theoretical basis for soil management and ecological restoration. Soil carbon-nitrogen dynamics and pH jointly shape microbial community diversity and structure, but their specific effects are regulated by vegetation type and litter chemical properties. Therefore, subsequent research will conduct multidimensional integrated experiments combining third-generation sequencing technology with scalable deep learning frameworks to achieve high-throughput, precise classification of functional genes in non-cultured microorganisms. This will elucidate the abundance and distribution characteristics of key ecological functional genes involved in processes such as lignin degradation and nitrogen cycling, thereby clarifying the mechanisms by which soil microbial community structure and function respond to environmental heterogeneity during forest succession.

## 5. Conclusions

Our study on the different vegetation types in the Greater and Lesser Khingan Mountains ecotone revealed how variation of primary tree species affected the microbial communities of understory soils. As expected, we observed relatively significant changes in the α- and β-diversities of the soil’s microbial communities. These changes were accompanied by differences in the relative abundances of specific bacterial and fungal taxons. The soil physicochemical properties affected the microbial community’s structure of different forests. The pH, NO_3_^−^-N, NH_4_^+^-N, MAOC, TN, TP, and DOC significantly influenced bacterial communities, while DOC and pH were the key environmental factors shaping soil fungal communities. Our findings provide an important insight into the stability of forest ecosystems in ecotone ecosystems in high-latitude coniferous forest ecosystems and make a valuable contribution to microbial ecology. It also plays a role in the subsequent exploration of how to protect existing forestry resources.

## Figures and Tables

**Table 1:** Physicochemical properties of soils in different forest types.

Sample	QM	BP	QB	LB	LG	Effect_Size	F_Stat_Sig
pH	5.34 ± 0.07 a	5.08 ± 0.05 b	4.86 ± 0.07 c	4.80 ± 0.05 c	4.79 ± 0.04 c	0.873	F(4,10) = 17.26 ***
SWC (%)	18.61 ± 1.25 b	14.93 ± 1.29 b	14.22 ± 1.18 b	18.18 ± 1.08 b	23.40 ± 2.01 a	0.731	F(4,10) = 6.78 **
NH_4_^+^-N (mg/kg)	10.47 ± 1.10 b	3.88 ± 0.53 d	5.68 ± 0.74 cd	8.08 ± 0.55 bc	11.45 ± 0.98 a	0.859	F(4,10) = 15.22 ***
NO_3_^−^-N (mg/kg)	0.67 ± 0.05 b	0.30 ± 0.05 c	0.11 ± 0.02 d	0.36 ± 0.05 c	1.19 ± 0.04 a	0.974	F(4,10) = 95.53 ***
TN (g/kg)	2.50 ± 0.11 c	4.02 ± 0.16 b	2.53 ± 0.11 c	3.62 ± 012 b	5.29 ± 0.15 a	0.969	F(4,10) = 78.65 ***
TP (g/kg)	0.79 ± 0.11 b	0.94 ± 0.08 b	0.65 ± 0.06 b	0.87 ± 0.06 b	1.37 ± 0.11 a	0.793	F(4,10) = 9.59 **
TC (g/kg)	45.14 ± 1.84 c	52.25 ± 1.64 b	35.28 ± 1.65 d	44.61 ± 1.50 c	64.81 ± 2.30 a	0.937	F(4,10) = 36.95 ***
DOC (mg/kg)	224.58 ± 7.15 b	209.08 ± 9.69 b	254.24 ± 8.48 a	184.22 ± 6.54 c	225.11 ± 4.05 b	0.826	F(4,10) = 11.85 ***
POC (mg/g)	37.10 ± 1.81 b	34.26 ± 1.99 b	21.78 ± 1.35 c	32.72 ± 1.53 b	51.16 ± 2.09 a	0.934	F(4,10) = 35.31 ***
MAOC (mg/g)	2.42 ± 0.57 ab	2.21 ± 0.15 b	3.13 ± 0.18 ab	3.35 ± 0.14 a	2.79 ± 0.16 ab	0.515	F(4,10) = 2.66 ns

Note: Values are presented as the mean ± standard error (*n* = 3). Different letters indicate significant differences between treatments (*p* < 0.05). (** *p* < 0.05; *** *p* < 0.01; ‘ns’ indicates no significant difference).

**Table 2:** Pearson’s correlation coefficients between soil parameters and microbial α-diversity indices.

Soil Physicochemical Parameters	Bacteria Shannon	Bacteria Chao 1	Fungi Shannon	Fungi Chao 1
SWC	0.284	0.21	−0.232	−0.204
DOC	−0.223	−0.693 **	−0.321	−0.554 *
NH_4_^+^-N	0.127	−0.246	−0.296	−0.232
NO_3_^−^-N	0.354	0.079	0.050	−0.057
TN	0.531 *	0.393	0.093	−0.100
TC	0.624 *	0.379	0.186	−0.082
TP	0.357	0.211	−0.125	−0.275
pH	−0.464	−0.286	−0.043	−0.064
POC	0.354	0.104	−0.025	−0.114
MAOC	−0.004	−0.032	−0.071	0.111

Note: Values represent Pearson’s correlation coefficients (r). Asterisks indicate statistical significance: * *p* < 0.05, ** *p* < 0.01.

## Data Availability

The data presented in this study are available on request from the corresponding author.

## Supplementary Materials

The following supporting information can be downloaded at: https://www.mdpi.com/article/10.3390/microorganisms13092107/s1. Table S1: Relative abundance of the most abundant bacterial phyla (top 10) present in five forests; Table S2. Relative abundance of the rare abundant bacterial phyla present in five forests; Table S3. Relative abundance of the most abundant fungal phyla (top 10) present in five forests; Table S4. Relative abundance of the rare abundant fungal phyla present in five forests; Table S5. Relative abundance of the most abundant bacterial genera (top 10) present in five forests; Table S6. Relative abundance of the rare abundant bacterial genera present in five forests; Table S7. Relative abundance of the most abundant fungal genera (top 10) present in five forests; Table S8. Relative abundance of the rare abundant fungal genera present in five forests.
