# Supplementary material for: Response of Soil Microbial Communities Between Different Vegetation Types in the Greater and Lesser Khingan Mountains Ecotone in Northeast China
Source: Microorganisms. 2025 Sep 9;13(9):2107. doi: 10.3390/microorganisms13092107 (PMC12472221; doi:10.3390/microorganisms13092107)
Supplement: Supplementary file 1 [file microorganisms-13-02107-s001.zip › microorganisms-3830318-supplementary.pdf]

Table S1. Relative abundance of the most abundant bacterial phyla (top 10) present in five forests.

Sample	QM	BP	QB	LB	LG
Proteobacteria	7864.0 ± 196.3 a	8063.6 ± 682.2 a	6631.3 ± 478.2 a	7181.3 ± 842.7 a	8007.6 ± 278.8 a
Acidobacteriota	6543.7 ± 577.1 a	6561.3 ± 252.6 a	7269.0 ± 551.2 a	5819.7 ± 352.2 a	6489.0 ± 137.3 a
Actinobacteriota	4810.6 ± 591.4 a	3763.3 ± 220.9 a	3096.3 ± 118.1 a	4077.3 ± 559.2 a	4283.6 ± 177.1 a
Chloroflexi	1982.7 ± 344.6 b	2953.3 ± 91.5 b	4575.6 ± 168.5 a	4360.6 ± 599.8 a	2356.3 ± 330.2 b
Verrucomicrobiota	1676.0 ± 167.8 a	1933.7 ± 497.3 a	1778.7 ± 276.6 a	1850.0 ± 483.5 a	1783.7 ± 105.8 a
Methylomirabilota	743.7 ± 131.5 a	671.7 ± 76.9 a	566.70 ± 46.3 a	527.0 ± 47.3 a	559.3 ± 48.3 a
Gemmatimonadota	498.3 ± 52.6 a	625.3 ± 52.1 a	559.00 ± 28.6 a	518.7 ± 56.4 a	656.7 ± 86.5 a
Bacteroidota	460.0 ± 54.1 a	335.7 ± 58.2 a	214.70 ± 20.4 a	557.3 ± 137.8 a	585.0 ± 20.8 a
Planctomycetota	374.7 ± 17.7 a	394.7 ± 60.9 a	487.30 ± 54.7 a	305.3 ± 74.3 a	458.7 ± 60.7 a
Myxococcota	299.7 ± 9.6 a	265.3 ± 13.4 a	261.33 ± 50.6 a	276.0 ± 45.6 a	308.0 ± 12.5 a

Table S2. Relative abundance of the rare abundant bacterial phyla present in five forests.

Sample	QM	BP	QB	LB	LG
Patescibacteria	214.3 ± 20.7 a	124.3 ± 11.8 a	161.3 ± 46.9 a	107.3 ± 17.8 a	187.7 ± 29.9 a
Firmicutes	299.3 ± 14.3 a	119.0 ± 9.3 b	83.0 ± 9.0 b	111.0 ± 3.3 b	111.3 ± 5.9 b
RCP2-54	118.0 ± 20.4 a	176.7 ± 22.2 a	150.3 ± 35.5 a	141.0 ± 10.3 a	117.0 ± 2.9 a
WPS-2	102.3 ± 18.9 b	80.0 ± 9.2 b	172.0 ± 4.7 a	97.0 ± 11.1 b	47.3 ± 5.1 b

Table S3. Relative abundance of the most abundant fungal phyla (top 10) present in five forests.

Sample	QM	BP	QB	LB	LG
Basidiomycota	24234.3 ± 556.3 a	24122.3 ± 772.5 a	23437.3 ± 1167.8 a	19846.7 ± 1791.5 b	21540.0 ± 1763.7 a
Ascomycota	8060.3 ± 687.4 a	8049.7 ± 431.5 a	9761.0 ± 1013.0 a	13497.7 ± 1545.1 a	13492.7 ± 1493.3 a
unclassified	3136.3 ± 948.8 a	340.3 ± 35.6 a	962.0 ± 211.3 a	1087.0 ± 81.7 a	630.3 ± 125.8 a
Mortierellomycota	805.0 ± 129.3 b	1103.7 ± 138.4 ab	1370.3 ± 253.8 ab	1736.0 ± 102.9 a	780.3 ± 65.4 b
Rozellomycota	146.0 ± 29.9 b	2777.3 ± 742.0 a	818.0 ± 142.2 b	579.6 ± 59.8 b	101.7 ± 15.8 b
Fungi_phy_Incertae_sedis	1012.3 ± 195.2 a	617.0 ± 157.9 a	732.7 ± 109.0 a	624.3 ± 115.1 a	868.3 ± 131.6 a
Mucoromycota	55.3 ± 4.4 ab	426.0 ± 72.5 a	359.7 ± 142.4 ab	49.3 ± 12.4 ab	33.0 ± 6.7 b
Chytridiomycota	4.0 ± 2.3 a	14.0 ± 5.6 a	7.3 ± 2.5 a	14.3 ± 0.8 a	6.7 ± 2.0 a
Glomeromycota	1.0 ± 0.6 c	4.7 ± 1.4 bc	6.7 ± 0.4 b	19.7 ± 1.1 a	1.0 ± 0.5 c
Entorrhizomycota	0.3 ± 0.1 a	0.0 ± 0.0 a	3.0 ± 1.7 a	1.7 ± 0.9 a	0.7 ± 0.3 a

Table S4. Relative abundance of the rare abundant fungal phyla present in five forests.

Sample	QM	BP	QB	LB	LG
Basidiobolomycota	0.0 ± 0.0 a	1.3 ± 0.8 a	0.0 ± 0.0 a	0.3 ± 0.2 a	2.3 ± 1.3 a
Olpidiomycota	2.7 ± 1.3 a	1.3 ± 0.8 a	0.0 ± 0.0 a	0.0 ± 0.0 a	0.0 ± 0.0 a
Kickxellomycota	0.0 ± 0.0 a	0.0 ± 0.0 a	0.0 ± 0.0 a	2.3 ± 1.4 a	1.3 ± 0.8 a
Zoopagomycota	1.0 ± 0.6 a	1.0 ± 0.6 a	1.0 ± 0.6 a	0.0 ± 0.0 a	0.7 ± 0.4 a
Blastocladiomycota	0.3 ± 0.2 a	0.0 ± 0.0 a	0.0 ± 0.0 a	0.0 ± 0.0 a	0.0 ± 0.0 a

Table S5. Relative abundance of the most abundant bacterial genera (top 10) present in five forests.

Sample	QM	BP	QB	LB	LG
Candidatus_Udaeobacter	1318.0 ± 152.3 a	1553.7 ± 425.6 a	1518.3 ± 248.1 a	1634.3 ± 612.1 a	1525.7 ± 90.5 a
Xanthobacteraceae	1253.0 ± 99.5 a	1390.3 ± 72.9 a	1250.0 ± 69.9 a	1459.3 ± 105.5 a	1455.3 ± 31.7 a
Acidobacteriales	1107.3 ± 71.6 b	1174.0 ± 95.2 b	2045.0 ± 307.4 a	1175.7 ± 179.2 b	923.3 ± 97.5 b
Subgroup_2	1176.7 ± 71.5 ab	1247.0 ± 72.3 ab	1745.3 ± 215.5 a	791.7 ± 144.1 b	1074.0 ± 213.4 b
c__AD3	714.7 ± 164.3 b	1302.7 ± 91.7 b	2259.3 ± 234.7 a	1065.3 ± 196.2 b	588.0 ± 13.0 b
Vicinamibacterales	1185.0 ± 166.6 a	1017.0 ± 197.5 a	706.0 ± 69.5 a	961.0 ± 218.0 a	1410.7 ± 146.3 a
Gaiellales	1054.3 ± 207.9 a	1093.7 ± 47.1 a	879.3 ± 51.6 a	1164.7 ± 152.3 a	1042.0 ± 60.1 a
Elsterales	722.7 ± 6.8 a	1081.0 ± 242.1 a	1088.3 ± 117.1 a	820.7 ± 65.3 a	787.0 ± 66.8 a
Xanthobacteraceae	1114.3 ± 92.2 a	835.3 ± 94.8 a	746.0 ± 28.6 a	811.3 ± 106.7 a	931.0 ± 82.2 a
Candidatus_Solibacter	506.3 ± 17.7 a	761.7 ± 87.8 a	654.3 ± 63.6 a	852.3 ± 102.6 a	635.3 ± 33.9 a

Table S6. Relative abundance of the rare abundant bacterial genera present in five forests.

Sample	QM	BP	QB	LB	LG
Bradyrhizobium	857.0 ± 75.7 a	740.3 ± 67.1 a	659.3 ± 83.6 a	540.0 ± 75.4 a	592.0 ± 84.6 a
Mycobacterium	1188.3 ± 157.8 a	597.7 ± 115.1 b	468.0 ± 25.6 b	241.0 ± 52.7 b	612.3 ± 99.5 ab
Bryobacter	422.3 ± 36.2 a	799.0 ± 145.4 a	616.0 ± 78.9 a	609.3 ± 78.7 a	621.7 ± 28.9 a
Rokubacteriales	739.7 ± 129.8 a	668.7 ± 76.5 a	566.3 ± 46.2 a	516.0 ± 24.8 a	555.0 ± 49.4 a
IMCC26256	553.0 ± 20.5 ab	585.3 ± 29.8 ab	459.3 ± 38.2 b	551.3 ± 68.9 ab	749.7 ± 39.6 a

Table S7. Relative abundance of the most abundant fungal genera (top 10) present in five forests.

Sample	QM	BP	QB	LB	LG
Russula	7125.3 ± 946.8 a	6356.0 ± 929.2 a	7105.7 ± 578.8 a	1638.3 ± 460.1 a	3637.7 ± 872.6 a
Inocybe	987.7 ± 83.2 b	857.0 ± 140.2 b	926.7 ± 310.8 b	1070.7 ± 292.9 b	8723.7 ± 1012.6 a
Inosperma	94.0 ± 16.7 a	3945.3 ± 901.6 a	2315.0 ± 352.9 a	3875.3 ± 935.2 a	14.0 ± 3.1 a
Cortinarius	5785.0 ± 940.8 a	2010.0 ± 617.9 a	353.0 ± 97.9 a	246.0 ± 63.7 a	462.3 ± 95.3 a
Sebacina	2267.0 ± 634.6 a	339.0 ± 11.2 a	268.7 ± 88.4 a	2239.7 ± 534.9 a	2230.0 ± 436.7 a
unclassified_k__Fungi	3136.3 ± 848.8 a	340.3 ± 35.6 a	962.0 ± 211.3 a	1087.0 ± 81.7 a	630.3 ± 95.8 a
Oidiodendron	506.7 ± 38.0 a	503.3 ± 89.5 a	577.3 ± 123.9 a	1037.0 ± 180.3 a	2940.0 ± 815.8 a
Archaeorhizomyces	2183.3 ± 643.8 a	281.3 ± 49.2 a	549.3 ± 211.3 a	1064.7 ± 98.1 a	756.3 ± 173.7 a
Incertae_sedis	102.0 ± 28.9 b	2682.7 ± 347.5 a	710.0 ± 139.5 b	342.0 ± 58.8 b	28.0 ± 3.1 b
Fungi_gen_Incertae_sedis	1012.3 ± 195.2 a	617.0 ± 97.9 a	732.7 ± 109.9 a	624.3 ± 95.1 a	868.3 ± 91.6 a

Table S8. Relative abundance of the rare abundant fungal genera present in five forests.

Sample	QM	BP	QB	LB	LG
Humicolopsis	361.0 ± 90.4 a	1343.0 ± 204.1 a	759.7 ± 67.4 a	379.0 ± 86.0 a	363.3 ± 68.9 a
Lactarius	478.0 ± 94.6 a	568.7 ± 91.0 a	522.0 ± 91.9 a	1145.7 ± 189.2 a	367.0 ± 98.3 a
Saitozyma	213.0 ± 48.9 b	274.7 ± 54.2 b	670.0 ± 112.5 ab	1183.7 ± 246.9 a	739.0 ± 121.0 ab
Tomentella	383.3 ± 42.2 a	59.7 ± 17.4 a	311.7 ± 94.4 a	1971.7 ± 111.8 a	339.3 ± 89.7 a
Pseudosperma	30.0 ± 13.1 a	783.7 ± 141.1 a	1775.0 ± 304.7 a	1.3 ± 0.8 a	447.0 ± 92.5 a
